# From UBE3A to Angelman syndrome: a substrate perspective

**DOI:** 10.3389/fnins.2015.00322

**Published:** 2015-09-15

**Authors:** Gabrielle L. Sell, Seth S. Margolis

**Affiliations:** ^1^Department of Biological Chemistry, The Johns Hopkins University School of MedicineBaltimore, MD, USA; ^2^Solomon H. Snyder Department of Neuroscience, The Johns Hopkins University School of MedicineBaltimore, MD, USA

**Keywords:** Angelman syndrome, neurodevelopmental disorders, autism, ubiquitin ligase, UBE3A

## Abstract

Angelman syndrome (AS) is a debilitating neurodevelopmental disorder that is characterized by motor dysfunction, intellectual disability, speech impairment, seizures and common features of autism spectrum disorders (ASDs). Some of these AS related phenotypes can be seen in other neurodevelopmental disorders (Williams, 2011; Tan et al., 2014). AS patients commonly carry mutations that render the maternally inherited *UBE3A* gene non-functional. Duplication of the chromosomal region containing the *UBE3A* gene is associated with ASDs. Although the causative role for *UBE3A* gene mutations in AS is well established, a long-standing challenge in AS research has been to identify neural substrates of UBE3A, an E3 ubiquitin ligase. A prevailing hypothesis is that changes in UBE3A protein levels would alter the levels of a collection of protein substrates, giving rise to the unique phenotypic aspects of AS and possibly *UBE3A* associated ASDs. Interestingly, proteins altered in AS are linked to additional ASDs that are not previously associated with changes in *UBE3A*, indicating a possible molecular overlap underlying the broad-spectrum phenotypes of these neurogenetic disorders. This idea raises the possibility that there may exist a “one-size-fits-all” approach to the treatment of neurogenetic disorders with phenotypes overlapping AS. Furthermore, while a comprehensive list of UBE3A substrates and downstream affected pathways should be developed, this is only part of the story. The timing of when UBE3A protein functions, through either changes in UBE3A or possibly substrate expression patterns, appears to be critical for AS phenotype development. These data call for further investigation of UBE3A substrates and their timing of action relevant to AS phenotypes.

Angelman syndrome (AS) is a neurodevelopmental disorder that affects one in 15,000 individuals (Williams et al., 2006). Angelman syndrome is characterized by lack of speech, cognitive impairments, unusually happy demeanor, motor deficits and seizures, among other symptoms (Bird, 2014). Notably, Angelman syndrome shows symptomatic overlap with multiple other neurodevelopmental diseases, including Rett syndrome and Pitt-Hopkins syndrome (Forrest et al., 2013). While the phenotypic overlap of these diseases is discussed elsewhere (Tan et al., 2014; Margolis et al., 2015), it is an intriguing hypothesis to consider that converging molecular mechanisms contribute to a subset of their underlying symptoms.

Maternal loss of chromosomal region 15q11-13 is one cause of Angelman syndrome. While there are multiple genes in this region, it became apparent that mutations in *UBE3A*, a paternally-imprinted gene, are sufficient for causing Angelman syndrome (Kishino et al., 1997; Matsuura et al., 1997). Phenotype severity is correlated with the type of mutation, with the full deletion of 15q11-13 the most severe and point mutations in *UBE3A* less severe (Gentile et al., 2010; Valente et al., 2013). Moreover, while UBE3A is expressed off the maternal allele in mature neurons, it is biallelically expressed in most peripheral tissues, in glia, and in newly born neurons (Albrecht et al., 1997; Gustin et al., 2010; Judson et al., 2014). Despite this systemic reduction in UBE3A expression of AS individuals, much research has been focused on the central nervous system, ignoring peripheral contribution of reduced UBE3A expression to AS-associated phenotypes. Interestingly, chromosomal region 15q11-13 is found to be duplicated in 1–2% of all autism spectrum disorder (ASD) cases, providing additional evidence for the importance of this region in developing a functional nervous system (Cook et al., 1997; Sutcliffe et al., 1997). Indeed, duplications in the chromosomal region containing only UBE3A have been associated with developmental delay (Noor et al., 2015).

Mouse models with a maternally-inherited *Ube3a* deletion display many Angelman-like phenotypes, including learning and memory deficits, motor phenotypes, and seizures (Jiang et al., 1998; Miura et al., 2002). The phenotypes listed here are far from exhaustive, but have been reviewed elsewhere (Margolis et al., 2015). These phenotypes are only present when the deletion is maternally-inherited, with little to no phenotype in the paternally-deleted animals. In mouse models, not only are reductions in UBE3A protein expression capable of inducing neurological deficits, but duplications in UBE3A also show autism-like phenotypes, such as social and learning and memory deficits (Smith et al., 2011). The combination of mouse and human data suggests that UBE3A plays a fundamental and critical role in regulating pathways important for autism-like disorders.

UBE3A is an E3 ubiquitin ligase that functions to conjugate ubiquitin groups to a unique set of proteins (Scheffner et al., 1993; Huang et al., 1999). Ubiquitinated proteins are then, generally, targeted for degradation through the ubiquitin-proteasome system (Ciechanover and Schwartz, 1998). Since mutations in the catalytic domain of UBE3A are sufficient for development of Angelman syndrome (Kishino et al., 1997; Matsuura et al., 1997; Cooper et al., 2004), the lack of ubiquitination and degradation of UBE3A substrates is predicted to increase these substrate protein levels. Conversely, increases in UBE3A are expected to decrease levels of its substrates. It is hypothesized that this alteration in substrate levels contributes to the variety of phenotypes associated with AS and, potentially, ASDs.

Given the many neurological phenotypes associated with changes in UBE3A expression, one major task in the field has been to identify brain-derived targets as disease-relevant substrates. A previously published substrate of UBE3A is Pbl/ECT2, a RhoA guanine nucleotide exchange factor (RhoA GEF), although the contribution of AS phenotype has not been interrogated (Reiter et al., 2006). Another published substrate of UBE3A is the negative synaptic regulator Ephexin5, another RhoA GEF (Margolis et al., 2010). By reducing Ephexin5 in AS mice, a recent study found that Ephexin5 does not contribute to AS related cortical and cerebellar phenotypes such as vocalization deficits, seizure activity, or motor deficits (Mandel-Brehm et al., 2015). These results are not surprising considering that, in the brain, high Ephexin5 expression is restricted to hippocampus compared to surrounding brain regions when measured by *in situ* hybridization (Margolis et al., 2010). Another substrate reported recently is GAT1, a GABA transporter that is upregulated in the absence of UBE3A in the cerebellum. Treatment with THIP, a selective extrasynaptic GABA_A_ receptor agonist showed the capacity to rescue electrophysiological and motor deficits (Egawa et al., 2012). Arc, a cytoskeleton-associated protein known to regulate trafficking of AMPA receptors to the membrane, is reported to be a substrate of UBE3A (Greer et al., 2010). Consistent with Arc's role in contributing to AS related phenotypes recent data demonstrate that reduction of Arc levels is capable of ameliorating recovery time after audiogenic seizures without rescue of ultrasonic vocalizations or motor behavior deficits (Mandel-Brehm et al., 2015). Despite several groups having observed varying results regarding Arc's status as a direct substrate of UBE3A (Greer et al., 2010; Kühnle et al., 2013; Mabb et al., 2014; Mandel-Brehm et al., 2015), these data establish reduction in Arc levels are capable of mitigating symptoms. Given that these substrates contribute to only a subset of phenotypes associated with AS, they cannot be the sole enactors of phenotypic change in AS.

Despite this paucity of confirmed substrates, many non-substrates, i.e., proteins which are reported to not directly be regulated by UBE3A *in vivo* in mammalian tissue, have been shown to be altered in AS mouse model brains, either at the level of protein expression or activity, including α1-Na^+^/K^+^-ATPase and CaMKII phosphorylation (Weeber et al., 2003; van Woerden et al., 2007; Kaphzan et al., 2013; Mandel-Brehm et al., 2015). It should be noted that the drosophila homolog to α1-Na^+^/K^+^-ATPase was identified as an interactor with dUBE3A (Jensen et al., 2013), however this result was not replicated in mouse brain (Kaphzan et al., 2011). Rescue experiments modulating these misregulated proteins were done using genetic deletion of the target proteins or modification of CaMKII phosphorylation sites. In these cases, a subset of behavioral phenotypes, mostly related to learning and memory, were ameliorated (van Woerden et al., 2007; Kaphzan et al., 2013; Mandel-Brehm et al., 2015). Despite the amelioration of certain phenotypes, none of these studies reported rescuing all phenotypes observed in AS mouse models. While the molecular understanding for these disrupted pathways and their rescuing capacity remains to be fully elucidated, these data indicate that the development of AS can arise from a myriad of secondary and tertiary changes downstream of altering the expression of UBE3A (Figure 1). Interestingly, some of the proteins shown to be misregulated in AS and/or interact with UBE3A have also been shown to be disrupted in other neurodevelopmental disorders, including ASDs in which UBE3A is not reported to be misregulated (Table 1). These data raise the possibility that phenotypic overlap between AS and other neurodevelopmental disorders can be explained by the molecular overlap in downstream pathways important for nervous system development. Such overlapping molecular discoveries will be the first targets toward developing “one size fits all” type therapies with the potential of treating similar phenotypes of many neurodevelopmental disorders associated with AS.

**Figure 1:**
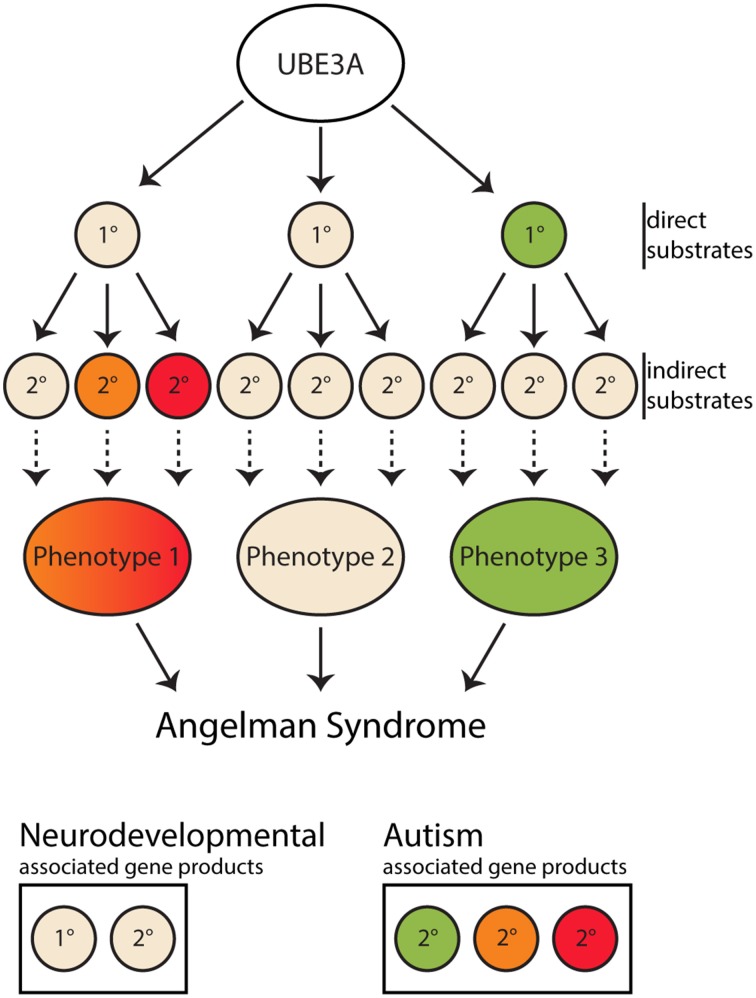
**Diagram of UBE3A substrate cascade to Angelman syndrome**. Angelman syndrome is caused by the pathological loss of maternal UBE3A protein expression, resulting in the loss of regulation of its pool of downstream substrates. UBE3A direct interactors/substrates are labeled as a 1° inside a circle. Indirect substrates/changes are labeled as 2° inside a circle. Gene products associated with neurodevelopment are in tan colored circles and associated with Autism spectrum disorders are colored. Following the loss of UBE3A changes to these 1°substrates are the initiators of cascade of events which lead to 2° changes. These 2° changes can be in the form of protein-protein interactions, cell biological, electrophysiological, etc. The convergence of these 2° and downstream changes produce phenotypes characteristic of Angelman syndrome. Given some proteins known to be misregulated in ASDs are primary or secondary interactors in this UBE3A-dependent pathway, as indicated by the colored substrates, the phenotypic overlap between Angelman Syndrome and autism spectrum disorders it is not surprising. Through the study of UBE3A substrates, more overlapping molecular changes underlying shared phenotypes may become apparent.

**Table 1:** **Possible mammalian UBE3A substrates**.

**Substrates/Interactors**	**Disease**	**Citation**
ANXA1	ASD	Shimoji et al., 2009; Correia et al., 2014
AR	ASD	Khan et al., 2006; Henningsson et al., 2009
Arc	AS, Fragile X	Park et al., 2008; Greer et al., 2010; Mandel-Brehm et al., 2015
CDKN1B	ASD	Mishra et al., 2009; Grey et al., 2013
DLG1	ASD	Matsumoto et al., 2006; Li et al., 2014
Ephexin5	Epilepsy	Margolis et al., 2010; Veeramah et al., 2013
ESR2	Asperger syndrome, ASD	Picard et al., 2008; Chakrabarti et al., 2009
Herc2	AS, ASD	Kühnle et al., 2011; Puffenberger et al., 2012; Harlalka et al., 2013
mGluR5	AS, Fragile X	Dölen et al., 2007; Pignatelli et al., 2014
SOD1	ASD	Mishra et al., 2013; Kovac et al., 2014
TSC1	AS, TS, ASD	Smalley, 1998; Sun et al., 2015
TSC2	AS, TS, ASD	Smalley, 1998; Zheng et al., 2008; Sun et al., 2015
UBE3A	AS, ASD	Schwarz et al., 1998; Nurmi et al., 2001

*AS, Angelman Syndrome; TS, Tuberous sclerosis; ASD, Autism spectrum disorder*.

While a comprehensive list of UBE3A substrates and downstream effected pathways should be developed, this is only part of the story. The functional interaction between UBE3A and these pathways will likely vary based on both the expression profile of UBE3A and the pathways in question.

Both the total protein level and subcellular localization of UBE3A is altered over development in neurons (Dindot et al., 2008; Williams et al., 2010; Judson et al., 2014). As previously mentioned, pyramidal cells in both cortex and hippocampus express UBE3A from the maternal allele. In intact brains, maternal UBE3A expression is much higher in mature neurons as opposed to newly born neurons, both early in development as well as in adult-born neurons (Dindot et al., 2008; Judson et al., 2014). Over time, this adult level of expression is reduced in the mammalian brain during advanced aging (Williams et al., 2010). Despite this robust age-related decrease in UBE3A expression, older individuals do not develop Angelman syndrome. This seemingly contrary idea highlights the possibility of separating UBE3A functions between the development of the CNS and in the adult CNS. Not only are levels of UBE3A consistently reported as changing across development, the localization of UBE3A shifts to the nucleus as development progresses. Maternal UBE3A-YFP shows a shift toward nuclear and synaptic localization later in development (Judson et al., 2014). To add another layer of complexity, neuronal activity in culture leads to increased localization of UBE3A to the nucleus and plasma membrane (Filonova et al., 2014). Since this change in localization is also activity-regulated, this indicates that UBE3A interaction with specific substrates will be activity-regulated as well. Due to neuronal activity and compartment-specific localization we suggest that sensitivity of each individual substrate to UBE3A will be altered based on these parameters, making it possible to miss functionally-relevant interactions in development- or activity-dependent scenarios. In fact, measurement of UBE3A's activity toward its substrates in these developmentally-restricted windows, in various compartments, and under alternative neuronal activity scenarios has not been done and could elucidate a more complex layer of regulation by UBE3A not yet appreciated.

The changes in UBE3A localization, expression, and possibly activity toward distinct targets over development hints that the timing of treatment in AS will be crucial to the success of the intervention. The importance of timing is further emphasized by experiments which re-express UBE3A at different times in development in AS mouse models: despite correcting the loss of UBE3A, the number and particular subset of phenotypes that are ameliorated is variable. It is difficult to ignore the potent role timing of UBE3A expression may be playing in neuronal development.

Recent work from the Elgersma lab has returned the wildtype UBE3A allele to a mouse model of AS at different times in development using an inducible Cre model (Silva-Santos et al., 2015). The work indicates that later and later UBE3A expression resulted in fewer rescued phenotypes. For instance, marble burying and rotarod deficits were returned to wild type levels when UBE3A was re-expressed at birth. However, UBE3A no longer rescued these phenotypes when expressed in adolescence or adulthood. Interestingly, despite the lack of behavioral rescue, LTP in the Schaffer collateral was reinstated in both juveniles and adults. A similar experiment to reactivate UBE3a expression utilized the mechanism of *UBE3A* imprinting to induce UBE3A expression from the paternal allele. The paternal allele is silenced by the expression of an antisense RNA (*UBE3A-ATS)* that is highly expressed to prevent overexpression of UBE3A. When this transcript is silenced, paternal expression is reinstated up to 90% of control level. Utilizing antisense oligonucleotides to disrupt the *UBE3A-ATS* and induce paternal expression of UBE3A protein, some phenotypes, such as fear conditioning and body weight, were ameliorated even upon treatment as juveniles. However, many phenotypes, including the robust and reproducible motor phenotypes and marble burying, were not rescued (Meng et al., 2014). Pharmacological methods of expressing paternal UBE3A, while successful at initiating protein expression, have not been characterized to ameliorate Angelman syndrome phenotypes *in vivo* (Huang et al., 2012; King et al., 2013; Powell et al., 2013).

These studies, among others, have illustrated the importance of the timing of UBE3A expression in the intact animal. While re-expression of UBE3A later in development is capable of positively impacting synaptic plasticity, it less effectively rescues behavioral deficits. Such results question whether the clinically relevant phenotypes in AS mice are those related to cellular or behavioral changes. Given the complex nature by which UBE3A affects nervous system development it is likely that studying UBE3A interactions and downstream effects relevant to AS pathogenesis can be best understood through *in vivo* studies. Systems such as neuronal culture are more apt to answer questions about general UBE3A function in the neuron, as developmental dependent UBE3A effects are stripped of meaning in context of *in vivo* AS pathogenesis.

UBE3A is a protein capable of mediating a variety of effects due to its consistent expression across many cell types and ages. However, while all functions are potentially important for fulfilling UBE3A's role in the body, only a subset of these functions is especially relevant to AS. Determining the latest possible point at which UBE3A re-expression is sufficient to rescue all phenotypes, including behavior, cell biology, and electrophysiology, will define the crucial window for UBE3A's function in AS. This window is the point in which UBE3A is formative for AS. Therefore, the large list of all UBE3A substrates can be narrowed to a short list of substrates altered in this time window and thus more likely to be AS-relevant. Perhaps more importantly, it is in this time window, and not in others, that research should be focused in order to determine the most disease-relevant pathways and mechanisms. This approach for studying UBE3A contribution to AS provides an opportunity to explore the overlap with other ASDs, as it is likely that the developmental restrictions on UBE3A's contribution to AS relevant substrates are similar to constraints present in other ASDs pathogenic proteins.

Mouse models of AS are an important tool for determining the UBE3A interactors and mechanisms in this critical window of AS development. As is well known, studying AS in mouse models is insufficient for complete understanding the human disease. In order for the UBE3A-dependent mechanisms elucidated in mice to have meaning, they must be translated from mouse to humans, both in respect to subtle changes in mechanism and to developmental age at which these mechanisms are occurring.

In humans, many changes likely occur from the loss of UBE3A at conception to the point at which an affected individual is diagnosed, and we are very much unaware of the true extent of these alterations. Therefore, a key question is: when is AS actually developing in humans? Work done in mouse models will give a framework for discussing these questions in the context of human AS, but the timing of human AS still needs to be established. Reports up to this point indicate that AS begins in early in life (Bird, 2014), and one hypothesis is that this early development of AS in mouse will translate to early development of AS in humans. Given that AS may be taking hold early in human development, early treatment will be crucial to efficacy of potential interventions. Therefore, improving methods of AS detection and removing the delay to diagnosis will be a key component to taking advantage of these mechanisms for the successful treatment of AS.

## Funding

The work in our laboratory is funded by following grants to SSM (R01 MH102364-02). GLS is supported by NSF Graduate Research Fellowship grant no. 1232825.

### Conflict of interest statement

The authors declare that the research was conducted in the absence of any commercial or financial relationships that could be construed as a potential conflict of interest.
